# Reactive Hypoglycemia From Metformin Immediate-Release Monotherapy Resolved by a Switch to Metformin Extended-Release: Conceptualizing Their Concentration-Time Curves

**DOI:** 10.7759/cureus.16112

**Published:** 2021-07-02

**Authors:** Ayesha Akram

**Affiliations:** 1 Internal Medicine, Combined Military Hospital, Rawalpindi, PAK; 2 Internal Medicine, Rawalpindi Medical University, Rawalpindi, PAK

**Keywords:** glycemic control, metformin immediate release, metformin extended release, therapeutic indications, adverse reactions, hypoglycemia

## Abstract

Metformin rarely, if ever, causes hypoglycemia when it is used as labeled. A 55-year-old woman presented to the medicine ward with an altered level of consciousness. She had been reviewed in an outpatient department three days earlier and prescribed 500 mg two times per day of metformin immediate-release (Met IR) for newly diagnosed type 2 diabetes mellitus (T2DM), to which she had been adherent; however, she had been experiencing intermittent episodes of hypoglycemia after taking the medication prescribed to treat her T2DM. On physical examination, she was diaphoretic and disoriented but responsive to sensory stimuli. In the ward, she received 25 ml of intravenous dextrose as the initial blood glucose reading was low at 54 mg/dl, and 4 ounces of apple juice additionally two hours later as her blood glucose level fell below 70 mg/dl again. She was no longer hypoglycemic a few hours later, and there was a significant neurological improvement. The remainder of the laboratory results, including serum renal and liver function tests, were normal. Met IR was discontinued, and metformin extended-release (Met XR) 500 mg/day was initiated at discharge. The patient's hypoglycemic episodes resolved within days after the initiation of Met XR. Hypoglycemia is rarely associated with accidental or suicidal overdose of metformin, metabolic dysfunction (e.g., renal insufficiency), exercise, missed meal, acute illness, or the initiation of additional antidiabetic medication. Albeit even uncommon, metformin-associated hypoglycemia may occur with no obvious trigger. In this context, we determine to what extent Met IR may contribute to the development of hypoglycemia in an individual case, but also that the risk could be mitigated by a switch to Met XR. In a preferred embodiment, the Met XR dosage form can be administered once a day, ideally with or after a meal, preferably with or after the evening meal, and it provides therapeutic levels of the drug throughout the day with peak plasma levels being obtained between four to eight hours after the administration (T_max_).

## Introduction

The term antidiabetic or antihyperglycemic drugs refers to drugs that are useful in managing type 2 diabetes mellitus (T2DM). Preferably, a first-line antidiabetic drug is a biguanide such as metformin or its pharmaceutically acceptable salt such as metformin hydrochloride. Metformin is an oral antihyperglycemic drug that improves glucose tolerance in T2DM by lowering both basal and postprandial plasma glucose [1]. Metformin exerts its metabolic effects by reducing hepatic gluconeogenesis due to the inhibition of mitochondrial glycerophosphate dehydrogenase, decreases hepatic lipogenesis by upregulation of AMP-activated protein kinase, and increases insulin-dependent peripheral glucose uptake and utilization [1,2]. In addition, it reduces appetite and decreases intestinal absorption of glucose [1]. The dosage of metformin is individualized on the basis of both effectiveness and tolerance in adults [1]. Previously, metformin was available as an immediate-release formulation (Met IR) only. Any such formulation is quickly absorbed and then rapidly cleared, necessitating multiple dosing with considerable plasma level fluctuations consisting of peaks and troughs. These fluctuations may account for transient side effects at the peak and a potential fall to subtherapeutic levels at the trough. This ultimately is the rationale behind developing a newer formulation, namely, metformin extended-release (Met XR) [3-5]. Met XR tablets are designed with the active ingredient overlaid with an enteric coat. The active drug is released through hydrated polymers, which expand the safe uptake of fluid, thereby prolonging gastric transit and delaying drug absorption in the upper gastrointestinal tract [1]. Owing to its slower absorption, Met XR can provide non-pulsating, therapeutic levels over a 12- to 24-hour period with less frequent administration, typically once daily [1]. Tolerability and even glycemic control in T2DM have been reported to improve with Met XR. Patient satisfaction and compliance are increased using a reduced dosing interval at one versus two or three times daily [3-5]. The bioavailability of the drug is not decreased by the presence of food, in fact a slight increase in the bioavailability is observed when the Met XR dosage form is administered with food [1]. Circulating levels of endogenous insulin are unchanged or slightly decreased by Met XR, and hence it carries a low risk of hypoglycemia [1]. Adverse events associated with metformin use are often gastrointestinal in nature, e.g., nausea, vomiting, and occasionally diarrhea [1]. However, hypoglycemia due to overdose, decreased drug clearance, nutritional deficits, multiple comorbidities, and drug-drug interactions, is rarely known to occur [6-11]. In this report, the case of a 55-year-old diabetic patient with no known comorbidity who developed metformin-associated hypoglycemia that promptly resolved after stopping Met IR and initiating Met XR is presented. A thorough comparison of the pharmacokinetics of Met IR and Met XR is undertaken in the Discussion section.

## Case presentation

A 55-year-old woman with newly diagnosed T2DM was found unresponsive by her family one morning. She had been seen in the outpatient setting three days ago and initiated on Met IR 500 mg twice daily in accordance with two consecutive fasting plasma glucose and hemoglobin A1c (HbA1c) readings of 126 and 155 mg/dl, and 6.3 and 6.8%, respectively, together with an insistence by the patient to "deliciously" control diabetes without an excessive cut-down on her favorite dessert. While in the ward, her daughter reported that the patient had experienced three episodes in the past two days characterized by headaches, nausea, sweating, and a feeling that she was going to pass out, despite adequate meals and adherence to Met IR as prescribed. During the most recent episode, her fingerstick glucose was 69 mg/dl and symptoms were relieved by oral fast-acting carbohydrates. Medical history included long-standing obesity only with a body mass index at present of 36 kg/m^2^. Of note, she had no history of renal insufficiency. When last checked at the outpatient, her urinary albumin-to-creatinine ratio had been 0.22 mg/mmol (normal value: <3.4). On presentation, she was diaphoretic and somnolent, with normal vital signs. Pupils were equal, round, and reactive to light. Further neurologic examination revealed disorientation to time and place but normal deep tendon reflexes and no focal sensory or motor deficits. The cardiopulmonary examination was normal. Her bedside point-of-care capillary blood glucose was checked and it was low, a finding confirmed by a serum blood glucose of 54 mg/dl. The diagnosis prompted intravenous administration of 25 ml of 50% dextrose. Emergent laboratory test results were unremarkable except for low blood glucose and an above-optimal low-density lipoprotein level (Table 1). There was no overt lactic acidosis on arterial blood gas results (pH: 7.4, PaCO_2_: 36 mmHg, and HCO_3_-: 24 mEq/L).

**Table 1:** Blood investigation results

Parameter	Value (reference)
Blood count	
Leukocyte count (×10^9^/L)	6.7 (4.5-11.0)
Erythrocyte count (×10^12^/L)	4.7 (3.5-5.5)
Hemoglobin (g/dL)	14.9 (12-16)
Hematocrit (%)	42 (36-46)
Mean corpuscular volume (fL)	85.4 (80-100)
Mean corpuscular hemoglobin (pg)	27.6 (25.4-34.6)
Mean corpuscular hemoglobin concentration (g/dL)	33.3 (31.5-34.5)
Platelet count (×10^9^/L)	240 (150-400)
Liver function tests	
Alanine aminotransferase (U/L)	22 (8-40)
Aspartate aminotransferase (U/L)	18 (8-40)
Alkaline phosphatase (U/L)	84 (45-115)
Albumin (g/L)	40 (35-50)
Total bilirubin (μmol/L)	4 (2-17)
Endocrine/miscellaneous	
Random blood glucose (mg/dL)	54 (hypoglycemia: ≤70)
Vitamin B12 (pmol/L)	253 (118-701)
Thyroid-stimulating hormone (mIU/L)	2.4 (0.5-5.0)
Renal function tests	
Urea (mmol/L)	4.4 (2.5-7.1)
Creatinine (μmol/L)	75 (53-106)
Serum electrolytes	
Sodium (mEq/L)	138 (136-145)
Potassium (mEq/L)	4.5 (3.5-5.0)
Chloride (mEq/L)	102 (95-105)
Bone profile	
Calcium (mmol/L)	2.3 (2.1-2.7)
Phosphate (mmol/L)	1.4 (1.0-1.5)
Coagulation profile	
Prothrombin time (seconds)	14 (11-15)
Partial thromboplastin time (seconds)	32 (25-40)
Lipid tests	
Cholesterol (mmol/L)	4.6 (3.9-6.2)
Triglycerides (mmol/L)	0.8 (0.4-1.6)
High-density lipoprotein (HDL) cholesterol (mmol/L)	1.2 (desirable: >1.3)
Low-density lipoprotein (LDL) cholesterol (mmol/L)	3.2 (optimal: <2.6, near/above optimal: 2.6-3.4)
Very-low-density lipoprotein (VLDL) cholesterol (mmol/L)	0.4 (0.1-1.7)

The patient’s condition improved transiently after the administration of an intravenous bolus of dextrose. However, repeat blood glucose two hours later fell to 67 mg/dl and 4 ounces of apple juice were given at this stage. Subsequently, levels began trending towards the normal range without any recurrence of hypoglycemia, reflected also by her conscious and oriented mental status, till hospital discharge. Serial measurements of blood glucose are documented in Table 2.

**Table 2:** Blood glucose levels

Time after the morning dose of metformin immediate-release (Met IR)	Blood glucose levels (mg/dL)
@0400	54 ➜ intravenous dextrose administered
@0415	115
@0600	67 ➜ one 4 oz. apple juice
@0800	85
@1100	110

Though not very familiar, the temporal association of hypoglycemia with the initiation of Met IR and the fact that there were no other factors to explore caused the medication to be withheld; her therapy was transitioned to Met XR 500 mg once a day. Over the next two weeks, a review of her self-monitored two-hour postprandial blood glucose showed levels of 125-165 mg/dl. Laboratory analysis at follow-up (day 14) revealed the following - fasting plasma glucose: 102 mg/dl, HbA1c: 5.9%. Met XR was now titrated up by 500 mg, i.e., 1000 mg once a day. After three weeks of therapy with Met XR (day 21), the patient is happy because of optimal glycemic control. She has experienced no more hypoglycemic attacks since the new therapy was started. A reasonable alternative is to reduce the dose of Met IR to 250 mg twice a day (the tablet can be cut in half), but this was not experimented on in this particular case.

## Discussion

Metformin is the preferred initial medication for most patients with T2DM due to its overall efficacy and lack of hypoglycemia when used in monotherapy. Overall, the available case reports also suggest that clinically significant hypoglycemia is uncommon in metformin monotherapy and unlikely to occur abruptly in the absence of any predisposing conditions [6-11]. Indeed, there is no previous report of this phenomenon occurring merely due to Met IR, as is evident in this patient by the normal renal and liver function tests, and the absence of risk factors for hypoglycemia that include prolonged fasting, exercise, acute illness, or addition of another antidiabetic [1]. A randomized, multi-center trial compared 24 weeks of treatment with twice-daily Met IR 1000 mg to once-daily Met XR 2000 mg in patients with T2DM and is probably the only one that cites reports of hypoglycemia in 1.1 % of patients exclusively on Met IR. Met XR was well tolerated at a single dose and there was three times less down-titration [12]. Metformin analogs are formulated with different pharmacokinetic properties, with the differences explained by a slower intestinal absorption of Met XR. Met XR provides a delayed mean time to maximum plasma concentration (T_max_), as compared to the T_max_ provided by Met IR. The delayed T_max_ occurs from four to eight hours after administration [1]. If Met XR is administered at dinner time (when dinner is normally eaten, generally between about 6 p.m. and 8 p.m.), the T_max_ would occur during the time when gluconeogenesis is expected to occur at its highest level [13]. The extent of absorption or systemic exposure is equivalent for both formulations, which is ideal especially in patients who switch to a Met XR formulation [1]. Peak plasma levels are about 20% lower as compared to a similar dose of Met IR [1]. In this respect, three parallel studies were conducted on 78 healthy subjects. A sample size of 26 subjects per group provided >80% statistical power to detect a difference in pharmacokinetic parameters between at least one Met XR group and Met IR. Every subject received a one-day dosing of either Met IR 1000 mg in the fed or fasting state or Met XR 750 mg in the fasting state. Both Met XR and Met IR displayed a similar half-life. The maximum plasma concentration (C_max_) of Met XR was much lower than that of Met IR (p<0.05). Additionally, Met XR was generally well-tolerated [14].

**Figure 1:**
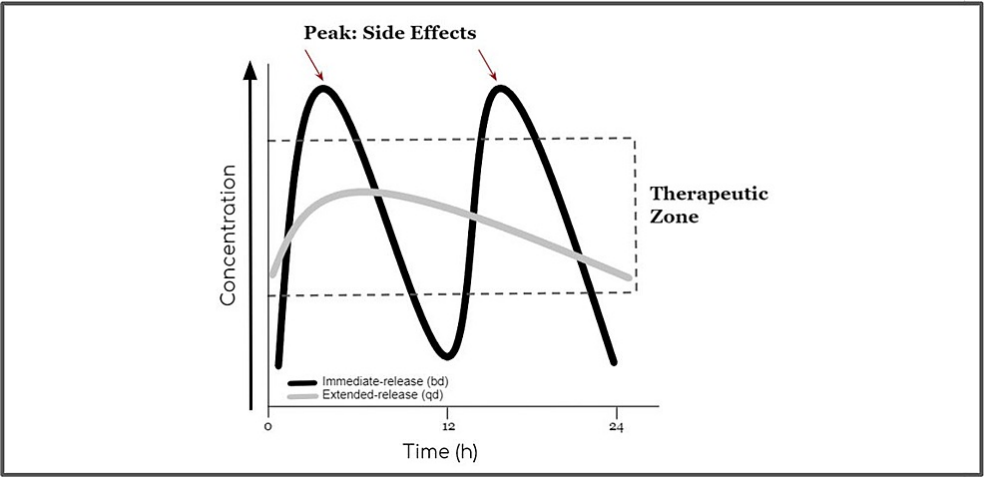
Simulated pharmacokinetic comparison of formulations of the same drug to compare immediate- and extended-release properties bd: twice daily; qd: once daily

The graph below (Figure 1) shows the plasma drug concentration-time curves of the two different oral formulations. The one represented by the gray curve shows a reduced and delayed peak level relative to the black curve, which is consistent with a sustained-release preparation. The fluctuation index - C_max_ minus minimum plasma concentration (C_min_)/average plasma concentration (C_avg_) - is of particular interest when comparing drugs with varying release properties. The lower the fluctuation index, the more likely the C_max_ is blunted while effectively extending the dosing interval, potentially allowing for up-titration to higher doses if needed for efficacy. This results in flatter concentration-time plots. There is even a benefit to having lower metformin levels in the blood during the afternoon in patients who are under concomitant therapy with at least one additional antidiabetic drug such as glyburide or its equivalents. In contrast to Met XR that does not increase endogenous insulin, a long-acting sulfonylurea, for instance, continues to stimulate pancreatic beta-cell insulin secretion even when blood glucose levels are normal (glucose-independent insulin release) and is therefore prone to causing hypoglycemia [1,15].

Some degree of research has been performed in the area of controlled-release compositions that employ antihyperglycemic drugs. The value of Met XR is further indicated by the results of a randomized, double-blind, active-controlled, six-month clinical trial among adult patients with drug-naive T2DM and HbA1c levels >7.0% and <8.5% [3]. Metformin was given in a randomized sequence at the maximum tolerated dose as Met IR in one group and as Met XR in the other. The efficacy endpoint was glycemic control as determined by the reduction in HbA1C, fasting plasma glucose, and postprandial plasma glucose levels after six months. Accordingly, insulin resistance was also measured. Met XR produced not only a statistically significant and sustained reduction in these measures of glycemic control compared with baseline (p<0.01) but also a more efficacious lowering of levels than Met IR (p<0.05). Secondly, the reduction in insulin resistance was consistent with these changes (p<0.01 vs. baseline, p<0.05 vs. Met IR). Of note, there was no significant increase in adverse events with an average 1000 ± 500 mg/day dose of Met XR, even with improved efficacy at this dose. Although there were no cases of hypoglycemia in either group, the overall incidence of gastrointestinal side effects was lower among those who received Met XR. While there may be other explanations for the improved outcome on Met XR, including enhanced compliance due to less frequent dosing, minimizing fluctuations in serum drug levels or continued glycemic control is likely to have played an important role [3]. In the present case too, Met XR achieved "therapeutically effective reduction", which signifies that plasma glucose levels were reduced by an effective amount compared to a Met IR reference standard when the dosage form was orally administered to the patient on a once-daily basis. Further contemplated for the purpose of the present case is that Met IR persisted at its peak plasma levels for approximately two hours, although this claim is directed to the pharmacokinetic parameters of an individual patient. A limitation of this study is that serum metformin levels were not determined. The Naranjo algorithm, a tool used to discern an adverse drug reaction, was used retrospectively to analyze this case presentation. According to the algorithm, this case showed a score of 7, which coincides with a "probable" diagnosis of adverse drug reaction. This is supported by clinical improvement and resolution of hypoglycemic symptoms with the discontinuation of Met IR. This patient had symptomatic hypoglycemia, confirmed by the Whipple triad [16], and is by definition "severe hypoglycemia" or "documented symptomatic hypoglycemia" [17].

## Conclusions

In accordance with the dosage forms of the present case, it has been determined that this patient suffering from T2DM achieved improved results (e.g., effectively lowered blood glucose levels, lower risk of hypoglycemia) with Met XR than Met IR administered according to popular protocols, e.g., on a twice-a-day basis. Another clear advantage is a reduction in the frequency of administration with Met XR. All of these findings suggest that Met XR may improve the quality of therapy and the safety profile relative to a conventional dosage form in patients with T2DM.
